# The impact of caring for COVID-19 patients on nurse professional identity: A cross-sectional study using propensity score analysis

**DOI:** 10.3389/fpubh.2022.1066667

**Published:** 2022-11-29

**Authors:** Lai Kun Tong, Ming Xia Zhu, Si Chen Wang, Pak Leng Cheong, Iat Kio Van

**Affiliations:** ^1^Research Management and Development Department, Kiang Wu Nursing College of Macau, Macao SAR, China; ^2^Education Department, Kiang Wu Nursing College of Macau, Macao SAR, China

**Keywords:** nurse, professional identity, propensity score matching, COVID-19 patient, COVID-19 pandemic

## Abstract

**Objective:**

To examine the impact of caring for COVID-19 patients on the professional identity of nurses.

**Methods:**

An online survey was conducted between 19 May and 7 August 2020 in 11 Chinese cities, including Dongguan, Foshan, Guangzhou, Hong Kong, Huizhou, Jiangmen, Macao, Shenzhen, Zhaoqing, Zhongshan, and Zhuhai. Propensity score matching was used to adjust for confounding variables between nurses with and without experience caring for COVID-19 patients. To analyze the impact of caring for COVID-19 patients on nurses' professional identity, a nominal logistic regression model was used rather than an ordinal regression model because the parallel regression assumption was violated.

**Results:**

After propensity score matching, the final sample contained 1,268 participants, including 634 nurses who cared for COVID-19 patients. During the COVID-19 outbreak, 88.6% of nurses had high levels of professional identity. Nurses who cared for COVID-19 patients had the lowest percentage of high score level on the professional identity subscale for “sense of organizational influence,” as did nurses who did not care for COVID-19 patients. The findings indicated that nurses who cared for COVID-19 patients were 17.95 times more likely to have a high professional identity than a low professional identity (95% CI 2.38–135.39, *p* = 0.005), after completely controlling for the other factors. There were significant differences between nurses who cared for COVID-19 patients and those who did not in scores on the subscales of professional identity, except for the subscales “sense of self-decision-making” (χ^2^ = 4.85, *p* = 0.089) and “sense of organizational influence” (χ^2^ = 4.71, *p* = 0.095).

**Conclusion:**

Nurses' professional identity is positively impacted by their experience caring for COVID-19 patients. Caring for COVID-19 patients should be highlighted as an opportunity to enhance nurses' professional identity. To further enhance the professional identity of nurses, we call for visible nursing leadership during the COVID-19 pandemic and improve their working environment.

## Introduction

Coronavirus disease 2019 (COVID-19) puts additional stress on nurses as they deal with a variety of stressors in their daily work. A meta-analysis showed that the prevalence of stress, anxiety, depression, and sleep disturbance among nurses working during the COVID-19 epidemic was 43, 37, 35, and 43%, respectively (1). High-intensity and high stress jobs caused nurses to experience high levels of burnout during the COVID-19 pandemic (2). In addition, nurses are at risk of infection and death during the COVID-19 pandemic. As of 31 December 2020, approximately 10% of those diagnosed with COVID-19 were health care workers, and COVID-19 has resulted in the deaths of 2,262 nurses in 59 countries (3). Nurses exposed to various stressors have increased levels of dissatisfaction with their jobs and are more likely to turnover. The turnover intention of nurses during COVID-19 was significantly higher than that before COVID-19 (4). It is estimated that there was a shortage of 5.9 million nurses before the COVID-19 outbreak (5). The COVID-19 pandemic is exacerbating the shortage of nurses.

Studies have confirmed that professional identity plays a critical role in nurse retention (6, 7). A recent study revealed that nurses with higher levels of professional identity were more likely to participate in the fight against COVID-19 (8). Professional identity is often defined as career, occupational or vocational identity (9) and refers to “one's professional self-concept based on attributes, beliefs, values, motives, and experiences” (10). Professional identity is an important issue for nurses because it is closely related to the nursing profession's unique nursing roles, responsibilities, values and ethical standards (11). An integrative literature review showed that the professional identity of nurses was influenced by three factors: the self, the role and the context, among which the context was the most important because the context of practice affected the other two factors (12). The professional identity of nurses is not invariable, but constantly develops and changes (13). The challenge of formulating professional identity has increased for nurses during the COVID-19 pandemic. What is the impact of the COVID-19 pandemic on nurses' professional identity? Existing studies of the professional identity of nurses during the COVID-19 pandemic have focused on status (14), influencing factors (15), and correlations with stress (14) and burnout (16). To our knowledge, studies on the professional identity of nurses caring for confirmed patients when major public health emergencies occur are lacking. Thus, this study is timely. Additionally, the number of nurses who will care for COVID-19 patients will continue to rise as the pandemic progresses. However, nurses who care for COVID-19 patients may experience negative emotions, including fatigue and helplessness, as a result of their intense work and fear, which can negatively impact their professional identity (17). To develop appropriate countermeasures, it is crucial to gain an in-depth understanding of the influence experienced in caring for patients with COVID-19 on nurses' professional identity. The aim of the study was to examine the level of nurses' professional identity during the COVID-19 pandemic and the impact of caring for COVID-19 patients on the professional identity of nurses.

Professional identity develops dynamically (18) and is influenced by a variety of factors, including the work environment (12), and public image (19). It has been reported that nurses' attitudes, experiences, and behaviors are influenced by the complexity, severity, proximity, and novelty of emerging infectious disease epidemic events (20). As a result, their professional identity may be positively or negatively affected (21). Both positive and negative effects of the COVID-19 pandemic were observed on nurses' professional identity, but overall, their professional identity rose during the pandemic (22, 23). Based on the results of previous studies, this study hypothesized that the COVID-19 pandemic had a positive impact on nurses' professional identity.

It is difficult to use an experimental design to study the influence of caring for confirmed patients on nurses' professional identity during major public health emergencies. It is unethical and infeasible to assign nurses to care for confirmed patients or not, since even nurses assigned to the non-confirmed group typically provide care for confirmed patients at uncertain times. Nurses caring for confirmed patients account for a small proportion of the total nurse population, and the characteristics of the two groups may differ considerably. As a statistical method for dealing with data from observational studies, propensity score matching is used to reduce the impact of data bias and confounding variables, allowing for a more reasonable comparison between the exposure group and the control group (15). In this study, the analysis was conducted using propensity score matching to correct for confounding variables, allowing objective analysis of the relationship between the experience of caring for COVID-19 patients and professional identity.

## Methods

### Study design, setting and participants

A cross-sectional survey was conducted among 8,065 nurses between 19 May and 7 August 2020 in 11 Chinese cities, including Dongguan, Foshan, Guangzhou, Hong Kong, Huizhou, Jiangmen, Macao, Shenzhen, Zhaoqing, Zhongshan, and Zhuhai. This study was conducted during the COVID-19 pandemic, which was declared a global pandemic in March 2020 (24). In 2019, these 11 cities accounted for 11.4% of China's gross domestic product (25). There were 258,364 nurses in these 11 cities, which represented 6.3% of the total number of nurses in China (26). The sample size was calculated using an online sample size calculator (http://www.raosoft.com/samplesize.html), assuming a 50% response distribution, a 99% confidence interval, and a 3% margin of error. For added contingency, the minimum sample size (*n* = 1,688) was increased by 20% to *n* = 2,026.

Convenience sampling was used to recruit nurses who met the following inclusion and exclusion criteria. The inclusion criteria were as follows: (1) nurses working in the above 11cities and (2) nurses with Chinese language skills. The exclusion criteria were as follows: (1) nurses who were trainees; (2) nurses who were currently on probation; (3) nurses who had come out of retirement to provide temporary support; and (4) nurses who refused to participate.

The survey was conducted online using the Wenjuanxing e-questionnaire platform (Wenjuan Xing Tech Co. Ltd., Changsha, China), which is widely used in China. The poster containing the QR code of the survey was pushed through WeChat, the most popular social networking app in China, to nurses across 11 cities. To promote this study effectively, the research team invited hospitals, nursing professional groups, and nursing colleges in 11 cities to assist. After reading the informed consent, participants clicked the “Agree” button before answering the questionnaire. For anonymity purposes, no personal information, such as name or contact information, was collected in the questionnaire. To improve the quality of the data, each device was set to answer the questionnaire once, and questionnaires with response times below 90 s were excluded from the study.

**Figure 1:**
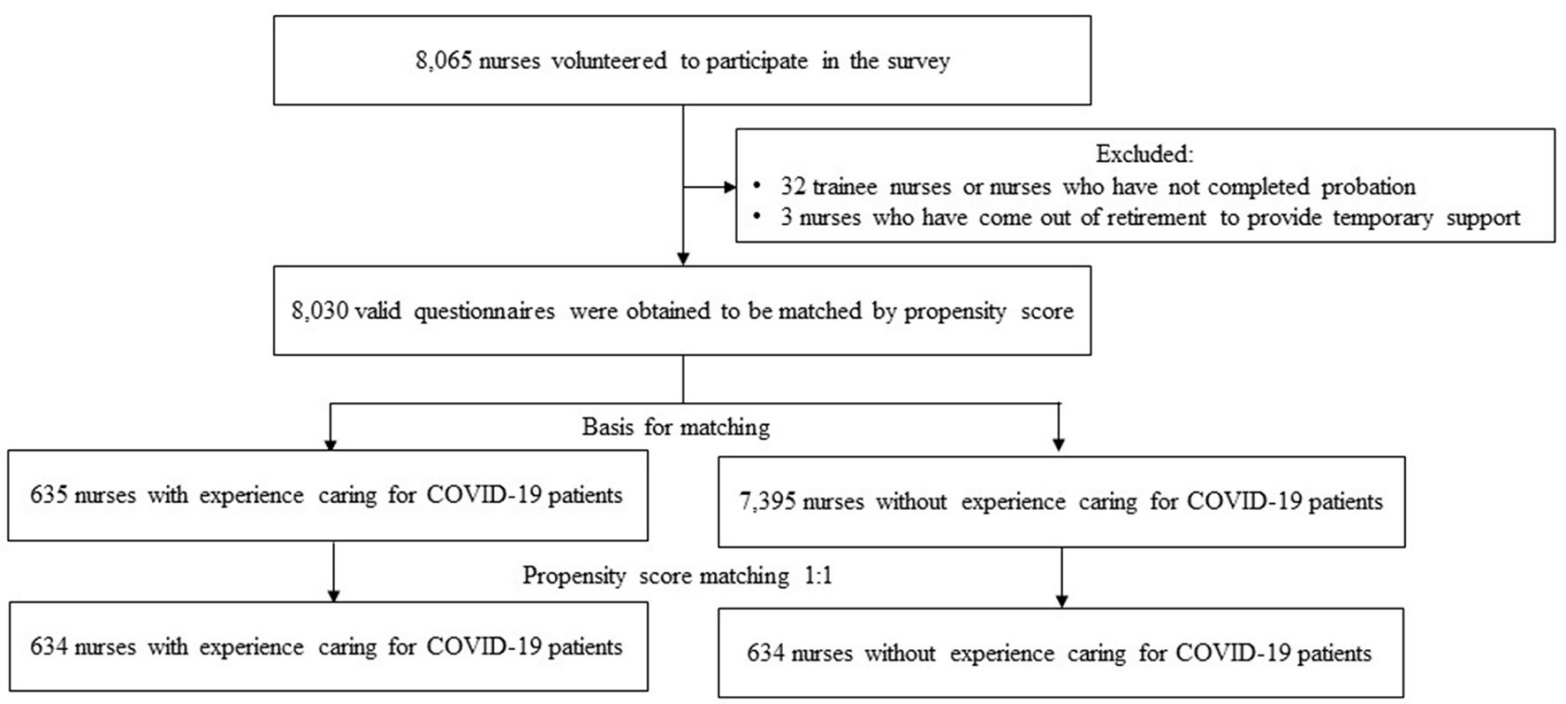
Flowchart of nurses inclusion.

A total of 8,065 questionnaires were collected in this study. Further screening of the questionnaires was performed according to the exclusion criteria, and 8,030 nurses were entered into the matching baseline (Figure 1).

### Questionnaire

#### Demographic characteristics

Data on participants' demographic information were collected, including gender, age, education background, marital status, number of children, years of working as a nurse, years of working at the current company, professional title, whether they were administrative supervisors, whether they were clinical teaching teachers, and the sufficiency of their ward workforce.

#### The experience of caring for COVID-19 patients

It was measured by asking “Did you care for COVID-19 patients during the COVID-19 outbreak?”

#### The nurse's career identity scale

Professional identity can be measured with a variety of screening tools. According to a systematic review of professional identity screening tools, the Macleod Clark Professional Identity Scale is the most effective tool for a range of health professions, while the Nurses' Professional Values Scale-Revised (NPVS-R) is the most effective tool for nurses (27). The Chinese (Taiwanese) version of the NPVS-R showed satisfactory reliability and validity. As Chinese from Taiwan use the traditional written system with complicated strokes, whereas Chinese from mainland China use the simplified written system with fewer strokes, applying the Chinese (Taiwanese) version applied to mainland Chinese may not be appropriate.

The nurse's career identity scale developed by Asians was translated into simplified Chinese and widely applied to nurses in mainland China, so this study used the scale. The nurse's career identity scale contains 21 items that are rated from 1 (completely disagree) to 7 (completely agree), with a score ranging from 1 to 7 to measure nurses' professional identity (28). It consists of seven subscales: (1) sense of control; (2) sense of agreement; (3) sense of meaning; (4) sense of self-potency; (5) sense of self-decision-making; (6) sense of organizational influence; and (7) sense of patient influence. The simplified Chinese version of the scale was considered valid and reliable with a content validity index of 0.92 and a Cronbach's alpha of 0.84 (29). Participants were divided into low (score < 3), medium (score 3–5), and high (score > 5) professional identities according to their scores. Obtaining authorization for the use of the scale was done at the beginning of the study.

### Data analysis

Pearson's χ^2^ test or Fisher's exact test was employed to examine associations between demographic characteristics and experience of caring for COVID-19 patients.

Propensity score matching has been widely used in observational studies and nonrandomized clinical trials to make data for experimental and control groups comparable in retrospective analyses. In the present study, propensity score matching was used to adjust for confounding variables between nurses with and without experience of caring for COVID-19 patients to draw more accurate conclusions about the association between experience of caring for COVID-19 patients and professional identity. Two steps are involved in propensity score matching. First, logistic regression was used to estimate the propensity score. The variables used in the propensity score included gender, age, education background, marital status, number of children, years of working as a nurse, years of working at the current company, professional title, whether they were administrative supervisors, whether they were clinical teaching teachers, and the sufficiency of their ward workforce. The second step is the matching procedure. Propensity score matching was performed at 1:1 with a caliper value of 0.02 using a logistic regression. A sufficient number of controls is one of the most important factors for ensuring quality in matching procedures. It has been suggested that a factor over 4:1 (control group: experimental group) is acceptable (30), whereas in the current study the factor was larger than 11:1 (7,395:635; nurses with experience of caring for COVID-19 patients: nurses without experience of caring for COVID-19 patients).

Pearson's χ^2^ test or Fisher's exact test was used to examine associations between demographic characteristics and professional identity categories. To analyze the impact of caring for COVID-19 patients on nurses' professional identity, a nominal logistic regression model was used rather than an ordinal regression model because the parallel regression assumption was violated. If a significant difference existed in nominal logistic regression between difference experiences of caring for COVID-19 patients, Pearson's χ^2^ test or Fisher's exact test was used to analyze the relationship between the score level of seven subscales of professional identity and experience of caring for COVID-19 patients and the significance level was adjusted using the Bonferroni method. Data were analyzed using SPSS 22.0, and statistical significance was set at *p* < 0.05.

## Result

### Characteristics of participants

This study eventually included 8,030 nurses. The majority of participants were women (96.6%), and more than half of them had a bachelor's degree or above. Fewer than 8% of respondents had experienced caring for COVID-19 patients (Table 1). According to chi-square test results, there was a significant difference in gender, education background, marital status, number of children, years of working as a nurse, years of working at the current company, whether they were administrative supervisors, and whether they were clinical teaching teachers between nurses with and without caring for COVID-19 patients, but no significant difference was observed in age, professional title, or the sufficiency of their ward workforce (Table 1).

**Table 1:** Characteristics of participants before and after propensity score matching.

	**Before matching (*****N*** = **8,030)**				**After matching (*****N*** = **1,268)**			
	**The experience of caring for COVID-19 patients**		**χ^2^**	***p***	**The experience of caring for COVID-19 patients**		**χ^2^**	***p***
	**Yes *n* (%)**	**No *n* (%)**			**Yes *n* (%)**	**No *n* (%)**		
Overall	635 (7.9)	7,395 (92.1)			634 (50.0)	634 (50.0)		
Gender			35.977	< 0.001			0.100	0.751
Male	48 (7.6)	587 (7.9)			47 (7.4)	50 (7.9)		
Female	226 (92.4)	7,169 (92.1)			587 (92.6)	584 (92.1)		
Age (years)			2.546	0.280			0.924	0.630
≤ 30	345 (54.3)	3,851 (52.1)			345 (54.4)	361 (56.9)		
31–50	279 (43.9)	3,352 (45.3)			278 (43.8)	264 (41.6)		
≥51	11 (1.7)	192 (2.6)			11 (1.7)	9 (1.4)		
Education background			14.707	0.001			0.922	0.631
Diploma	243 (38.3)	3,411 (46.1)			243 (38.3)	247 (39)		
Graduate	381 (60.0)	3,859 (52.2)			380 (59.9)	380 (59.9)		
Postgraduate	11 (1.7)	125 (1.7)			11 (1.7)	7 (1.1)		
Marital status			21.442	<0.001			2.577	0.276
Single	269 (42.4)	2,472 (33.4)			268 (42.3)	279 (44)		
Married	352 (55.4)	4,775 (64.6)			352 (55.5)	348 (54.9)		
Other	14 (2.2)	148 (2)			14 (2.2)	7 (1.1)		
Had one or more children			18.020	<0.001			0.079	0.779
Yes	325 (51.2)	4,423 (59.8)			325 (51.3)	320 (50.5)		
No	310 (48.8)	2,972 (40.2)			309 (48.7)	314 (49.5)		
Years of working as a nurse			8.333	0.016			1.155	0.561
≤10	398 (62.7)	4,309 (58.3)			397 (62.6)	414 (65.3)		
11–20	165 (26.0)	1,948 (26.3)			165 (26)	157 (24.8)		
≥21	72 (11.3)	1,138 (15.4)			72 (11.4)	63 (9.9)		
Years of working at the current company			8.132	0.017			0.505	0.777
≤10	456 (71.8)	5,033 (68.1)			455 (71.8)	466 (73.5)		
11–20	134 (21.1)	1,580 (21.4)			134 (21.1)	127 (20)		
≥21	45 (7.1)	782 (10.6)			45 (7.1)	41 (6.5)		
Professional title			1.201	0.273			1.324	0.250
Junior	426 (67.1)	5,116 (69.2)			426 (67.2)	445 (70.2)		
Intermediate and senior	209 (32.9)	2,279 (30.8)			208 (32.8)	189 (29.8)		
Administrative supervisor			4.546	0.033			1.177	0.278
Yes	89 (14.0)	829 (11.2)			89 (14)	76 (12)		
No	546 (86.0)	6,566 (88.8)			545 (86)	558 (88)		
Clinical teaching			6.670	0.010			0.029	0.864
Yes	259 (40.8)	2,637 (35.7)			259 (40.9)	262 (41.3)		
No	376 (59.2)	4,758 (64.3)			375 (59.1)	372 (58.7)		
Workforce			2.128	0.345			0.036	0.982
Enough	152 (23.9)	1,860 (25.2)			151 (23.8)	149 (23.5)		
Barely enough	266 (41.9)	2,880 (38.9)			266 (42)	265 (41.8)		
Not enough	217 (34.2)	2,655 (35.9)			217 (34.2)	220 (34.7)		

After propensity score matching, the final sample contained 1,268 participants, including 634 nurses who cared for COVID-19 patients and 634 nurses who did not (Figure 1). After propensity score matching, no significant differences were found in gender, age, education background, marital status, number of children, years of working as a nurse, years of working at the current company, professional title, whether they were administrative supervisors, whether they were clinical teaching teachers, and the sufficiency of their ward workforce between nurses with and without caring for COVID-19 patient experience (Table 1), indicating a good match.

### Professional identity

In this study, 88.6% of nurses during the COVID-19 outbreak had high levels of professional identity. There was no significant difference in the level of professional identity among nurses by gender, age, education background, years of working as a nurse, or whether they were clinical teaching teachers (*p* > 0.05). Nurses with low professional identity were more likely to be married (*p* = 0.047), have children (*p* = 0.005), have worked at their current company for ≤10 years (*p* = 0.042), hold junior professional titles (*p* = 0.035), not serve as administrative supervisors (*p* = 0.036), work in organizations with enough staffing (*p* = 0.017), and not have any experience caring for COVID-19 patients (*p* < 0.001) than those with moderate or high professional identity (Table 2).

**Table 2:** Characteristics of participants according to the categorized professional identity after propensity score matching.

	**All, *n* (%)**	**Low professional identity, *n* (%)**	**Moderate professional identity, *n* (%)**	**High professional identity, *n* (%)**	**χ^2^**	***p***
Overall	1,268 (100)	19 (1.5)	126 (9.9)	1,123 (88.6)		
Gender					0.535	0.765
Male	97 (7.6)	2 (10.5)	8 (6.3)	87 (7.7)		
Female	1,171 (92.4)	17 (89.5)	118 (93.7)	1,036 (92.3)		
Age (years)					6.531	0.163
≤30	706 (55.7)	11 (57.9)	82 (65.1)	613 (54.6)		
31–50	542 (42.7)	7 (36.8)	42 (33.3)	493 (43.9)		
≥51	20 (1.6)	1 (5.3)	2 (1.6)	17 (1.5)		
Education background					7.183	0.127
Diploma	490 (38.6)	11 (57.9)	50 (39.7)	429 (38.2)		
Graduate	760 (59.9)	8 (42.1)	76 (60.3)	676 (60.2)		
Postgraduate	18 (1.4)	0 (0)	0 (0)	18 (1.6)		
Marital status					9.615	0.047
Single	547 (43.1)	9 (47.4)	70 (55.6)	468 (41.7)		
Married	700 (55.2)	10 (52.6)	54 (42.9)	636 (56.6)		
Other	21 (1.7)	0 (0)	2 (1.6)	19 (1.7)		
Had one or more children					10.538	0.005
Yes	645 (50.9)	11 (57.9)	47 (37.3)	587 (52.3)		
No	623 (49.1)	8 (42.1)	79 (62.7)	536 (47.7)		
Years of working as a nurse					7.861	0.097
≤10	811 (64)	11 (57.9)	94 (74.6)	706 (62.9)		
11–20	322 (25.4)	6 (31.6)	24 (19.0)	292 (26.0)		
≥21	135 (10.6)	2 (10.5)	8 (6.8)	125 (11.1)		
Years of working at the current company					9.936	0.042
≤10	921 (72.6)	12 (63.2)	105 (83.3)	804 (71.6)		
11–20	261 (20.6)	6 (31.6)	16 (12.7)	239 (21.3)		
≥21	86 (6.8)	1 (5.3)	5 (4.0)	80 (7.1)		
professional title					6.683	0.035
Junior	871 (68.7)	14 (73.7)	99 (78.6)	758 (67.5)		
Intermediate and senior	397 (31.3)	5 (26.3)	27 (21.4)	365 (32.5)		
Administrative supervisor					6.656	0.036
Yes	165 (13.0)	2 (10.5)	8 (6.3)	155 (13.8)		
No	1,103 (87.0)	17 (89.5)	118 (93.7)	968 (86.2)		
Clinical teaching					2.449	0.294
Yes	521 (41.1)	9 (47.4)	44 (34.9)	468 (41.7)		
No	747 (58.9)	10 (52.6)	82 (65.1)	655 (58.3)		
Workforce					12.051	0.017
Enough	300 (23.7)	7 (36.8)	22 (17.5)	271 (24.1)		
Barely enough	531 (41.9)	7 (36.8)	44 (34.9)	480 (42.7)		
Not enough	437 (34.5)	5 (26.3)	60 (47.6)	372 (33.1)		
The experience of caring for COVID-19 patients					17.783	<0.001
Yes	634 (50.0)	1 (5.3)	72 (57.1)	561 (50.0)		
No	634 (50.0)	18 (94.7)	54 (42.9)	562 (50.0)		

### Impact of caring for COVID-19 patients on nurse professional identity

For the nominal regression, variables were selected that differed significantly between the three professional identity category groups in Table 2. As shown in Table 3, the findings indicated that nurses who cared for COVID-19 patients were 2.38–135.39 times more likely to have a high professional identity than a low professional identity (*p* = 0.005), after completely controlling for the other factors.

**Table 3:** Predictors of professional identity after propensity score matching (multivariable nominal regression).

	**Moderate professional identity vs. low professional identity**			**High professional identity vs. low professional identity**		
	**OR**	**95% CI**	***p***	**OR**	**95% CI**	***p***
**Marital status**						
Single = Ref						
Married	0.052	0.004, 0.672	0.052	0.049	0.004, 0.580	0.017
Other	0	0, 0	<0.001		0, 0	
**Had one or more children**						
Yes = Ref						
No	28.096	2.300, 343.253	0.009	19.878	1.805, 218.937	0.015
**Years of working at the current company**						
≤10 = Ref						
11–20	0.365	0.027, 4.977	0.450	0.517	0.046, 5.792	0.593
≥21	1.492	0.131, 16.950	0.747	1.458	0.160, 13.334	0.738
**Professional title**						
Junior	0.458	0.098, 2.151	0.322	0.469	0.112, 1.974	0.302
Intermediate and senior = Ref						
**Administrative supervisor**						
Yes	0.895	0.136, 5.895	0.908	1.516	0.273, 8.416	0.634
No = Ref						
**Workforce**						
Enough = Ref						
Barely enough	0.453	0.137, 1.498	0.195	0.521	0.176, 1.543	0.239
Not enough	0.235	0.065, 0.843	0.026	0.471	0.144, 1.543	0.214
**The experience of caring for COVID-19 patients**						
Yes	23.727	3.058, 184.102	0.002	17.954	2.381, 135.391	0.005
No = Ref						

OR, odds ratio; 95% CI, 95% confidence interval.

Nurses who cared for COVID-19 patients had the highest percentage of high score level on the professional identity subscale for “sense of self-potency,” followed by “sense of control,” and had the lowest percentage for “sense of organizational influence,” followed by “sense of self-decision-making,” as did nurses who did not care for COVID-19 patients (Table 4). The proportion of nurses who cared for COVID-19 patients with low score levels on the five subscales, including “sense of control,” “sense of agreement,” “sense of meaning,” “sense of self-potency,” and “sense of patient influence,” was significantly lower than those who did not. There was no difference in the two subscales of “sense of self-decision-making” and “sense of organizational influence” among nurses with different experiences in caring for COVID-19 patients (Table 4).

**Table 4:** Relationship between the score level of subscales of professional identity and experience of caring for COVID-19 patients.

	**The experience of caring for COVID-19 patients**		**χ^2^**	***p***
	**Yes *n* (%)**	**No *n* (%)**		
Sense of control			13.032	0.001
Low	2 (0.3)_a_	18 (2.8)_b_		
Moderate	55 (8.7)_a_	52 (8.2)_a_		
High	577 (91.0)_a_	564 (89.0)_a_		
Sense of agreement			12.383	0.002
Low	3 (0.5)_a_	17 (2.7)_b_		
Moderate	80 (12.6)_a_	61 (9.6)_a_		
High	551 (86.9)_a_	556 (87.7)_a_		
Sense of meaning			17.056	<0.001
Low	3 (0.5)_a_	20 (3.2)_b_		
Moderate	83 (13.1)_a_	58 (9.1)_b_		
High	548 (86.4)_a_	556 (87.7)_a_		
Sense of self-potency			14.444	0.001
Low	1 (0.2)_a_	17 (2.7)_b_		
Moderate	47 (7.4)_a_	47 (7.4)_a_		
High	586 (92.4)_a_	570 (89.9)_a_		
Sense of self-decision-making			4.847	0.089
Low	14 (2.2)	27 (4.3)		
Moderate	123 (19.4)	110 (17.4)		
High	497 (78.4)	497 (78.4)		
Sense of organizational influence			4.705	0.095
Low	35 (5.5)	34 (5.4)		
Moderate	217 (34.2)	182 (28.7)		
High	382 (60.3)	418 (65.9)		
Sense of patient influence			8.797	0.012
Low	8 (1.3)_a_	21 (3.3)_b_		
Moderate	123 (19.4)_a_	98 (15.5)_a_		
High	503 (79.3)_a_	515 (81.2)_a_		

Each subscript letter denotes a subset of the categories whose column proportions do not differ significantly from each other at the 0.05 level.

## Discussion

To our knowledge, this study is the first to examine the impact of caring for confirmed patients on nurses' professional identity during a major public health emergency. The results of this study showed that nurses' professional identity during the COVID-19 outbreak was high. This study found that nurses who cared for COVID-19 patients were more likely to have a high professional identity than those who did not.

Overall, our results reveal that nurses' professional identity was at a high level during the COVID-19 pandemic. Ren et al. (31) used the same tool to conduct a cross-sectional survey on the professional identity of nurses in China before the outbreak of COVID-19 (March–April 2018), and the study results showed that only 57.0% of nurses' professional identity was at a high level. The results of this study showed that 88.6% of nurses' professional identity was at a high level during the COVID-19 pandemic (May–August 2020). It is suggested that the COVID-19 pandemic has a positive impact on nurses' professional identity. An Italian study found similar results (32), while a Spanish study revealed both positive and negative effects of the COVID-19 pandemic on health care workers' professional identity (21). A scoping review found that during the initial stages of the pandemic, health care workers reported overwhelmingly negative emotions, which gradually was followed by increasing positive reports of the impact on their professional identity (33). As a result of this study, Chinese nurses' identity has been positively impacted by the COVID-19 pandemic, and two potential reasons can be attributed to this. First, while nurses have experienced exhaustion and fear in dealing with the COVID-19 challenge, seeing patients improve and the outbreak successfully contained has given them a deeper understanding and confidence in the nature of nursing (34). Second, the professional self-perception of nurses will affect the formation of their professional identity and the professional identity of nurses is influenced by the public (35). The public and the media strongly identify with the bravery, selflessness and value of nurses during the COVID-19 pandemic (36). Nurses were rewarded financially and honorably for their contributions to the epidemic, which made nurses feel their own value and social image improved (34, 37). Thus, the COVID-19 pandemic had a positive impact on nurses' professional identity. Researchers may explore ways to sustain the positive impact on nurses' professional identity caused by the COVID-19 pandemic in the future.

However, it is worth noting that nurses had the lowest score in “sense of organizational influence,” meaning nurses perceived that they had insufficient influence on the organization. In China, doctors are the main body of hospitals, and some managers think that nursing is an occupation to carry out doctors' orders (38), which gives nurses few opportunities to participate in hospital management (39). Therefore, the “sense of organizational influence” dimension has always been the lowest scoring for Chinese nurses (40). The COVID-19 pandemic has required more than ever teamwork among health care workers caring for COVID-19 patients. This blurred professional boundaries, particularly between doctors and nurses, who often switched roles when caring for patients (41). The importance of nurses in safeguarding the health and well-being of patients has been highlighted by their significant contribution to the fight against COVID-19, so nursing groups even call for “the need for visible nursing leadership during COVID-19” (42).

According to this study, nurses' professional identity is positively impacted by their experience caring for COVID-19 patients. One of the prevention and control measures for COVID-19 in China is that both suspected and confirmed patients need to be isolated and treated in designated hospitals (43). Government policy stated that designated isolation hospitals were not allowed to visit patients during the COVID-19 outbreak. Because of the isolation policy, patients can only be accompanied by health care workers, and their basic care and emotional support were mainly provided by nurses (34, 44), who were the middleman in maintaining the close relationship between relatives and patients (45). Nurses providing direct care to COVID-19 patients said they were “meeting patient care needs in new ways while staying safe” (46). Nurses working in isolation hospitals felt they received more respect and recognition during the pandemic. They thought patients were more cooperative than before, showing more respect for nurses and expressing their gratitude (47). Nurses who had worked in the COVID-19 ward for more than 2 months pointed out that they spent most of their time with patients; thus, they saw themselves at the forefront of the fight against the epidemic and felt like heroes (48). Nurses working in isolation hospitals said they gained additional expertise and skills while caring for COVID-19 patients, which helped in their self-growth and future nursing practice (34). In addition, nurses who did not provide direct patient care during the COVID-19 pandemic believed the label “healthcare hero” was most appropriate for direct care nurses who risked everything (49). Based on the above, nurses caring for COVID-19 patients were significantly improved in the “sense of control,” “sense of agreement,” “sense of meaning,” “sense of self-potency,” and “sense of patient influence.” Nursing's “sense of self-decision-making” and “sense of organizational influence” are not affected by the experience of caring for COVID-19 patients, which merits further investigation to develop better measures to enhance nurses' professional identity.

The findings of this study revealed that there was no difference in the “sense of self-decision-making” and “sense of organizational influence” among nurses with different experiences in caring for COVID-19 patients. Nurses caring for COVID-19 patients experienced high levels of stress, anxiety, depression, and burnout (2, 50). Burnout among nurses working in COVID-19 designated hospitals and COVID-19 wards was high and increased with the length of working time in these facilities (2). Therefore, nurses who cared for COVID-19 patients reported lower job satisfaction and higher turnover intention than those who did not (46, 51). Increased workloads and deteriorating working conditions have affected the health and well-being of nurses frontline nurses caring for COVID-19 patients. Therefore, the experience of caring for COVID-19 patients did not positively impact on nurses' sense of self-decision-making. To mitigate the negative impact of caring for COVID-19 patients on nurses' professional identity, health policy makers should take measures to control and prevent their mental disorders and improve their working environment.

Regarding the sense of organizational influence, nurses believed that the workplace culture in isolation hospitals had improved and that management's attitude toward nurses had improved. While the hierarchy of doctors and other health care professionals still exists, this situation has become less pronounced in isolated hospitals (47). It is important to note that this positive impact is not limited to isolated hospitals. The results from a study of Chinese nurses' perceptions of the impact of the COVID-19 pandemic showed that the COVID-19 pandemic appears to have had positive effects at the social and organizational levels (47). Therefore, there was no difference in the “sense of organizational influence” among nurses with different experiences in caring for COVID-19 patients.

There are some limitations to this study and the results must be interpreted with caution. First, this study was conducted in only 11 cities out of 293 in China, and convenience sampling was used, which affected the generalizability of the findings. Second, this study was designed as a cross-sectional study, but cross-sectional studies assessed exposure and outcomes at the same time, making it difficult to infer causality. The strength of this study is that the total sample size is large enough, and it covers low, medium and high income cities, thus achieving powerful statistical results. Moreover, the analysis was conducted using propensity score matching to correct for confounding variables.

## Conclusions

Chinese nurses' professional identity was at a high level during the COVID-19 pandemic. Nurses who cared for COVID-19 patients were more likely to have a high professional identity than those who did not. Nurses caring for COVID-19 patients demonstrated significantly better “sense of control,” “sense of agreement,” “sense of meaning,” “sense of self-potency,” and “sense of patient influence,” but there was no difference between nurses with different experiences caring for COVID-19 patients with regard to “sense of self-decision-making” and “sense of organizational influence.”

## Data availability statement

The data that support the findings of this study are available from the corresponding author upon reasonable request.

## Ethics statement

This research was approved by the Research Management and Development Department of Kiang Wu Nursing College of Macau (No. 2019APR01). Informed consent was obtained from all respondents before they began the online questionnaire. Taking part in the survey was completely anonymous, and participants may withdraw at any time.

## Author contributions

IV conceived the study and was responsible for project administration. LT interpreted the data, conducted an in-depth analysis, and wrote the manuscript. MZ, SW, and PC were responsible for data collection. All authors contributed to the article and approved the submitted version.

## Funding

The study was funded by the Macao Foundation (2964/DS/2019).

## Conflict of interest

The authors declare that the research was conducted in the absence of any commercial or financial relationships that could be construed as a potential conflict of interest.

## Publisher's note

All claims expressed in this article are solely those of the authors and do not necessarily represent those of their affiliated organizations, or those of the publisher, the editors and the reviewers. Any product that may be evaluated in this article, or claim that may be made by its manufacturer, is not guaranteed or endorsed by the publisher.

## References

[1] Al Maqbali M Al Sinani M Al-Lenjawi B. Prevalence of stress, depression, anxiety and sleep disturbance among nurses during the Covid-19 pandemic: a systematic review and meta-analysis. J Psychosom Res. (2021) 141:110343. 10.1016/j.jpsychores.2020.110343 33360329 PMC7831768

[2] Galanis P Vraka I Fragkou D Bilali A Kaitelidou D. Nurses' burnout and associated risk factors during the Covid-19 pandemic: a systematic review and meta-analysis. J Adv Nurs. (2021) 77:3286–302. 10.1111/jan.14839 33764561 PMC8250618

[3] International Council of Nurses. International Council of Nurses Covid-19 Update. Geneva: International Council of Nurses (2021). Available online at: https://www.icn.ch/sites/default/files/inline-files/ICN%20COVID19%20update%20report%20FINAL.pdf. (accessed July 10, 2021).

[4] Nashwan AJ Abujaber AA Villar RC Nazarene A Al-Jabry MM Fradelos EC. Comparing the impact of Covid-19 on nurses' turnover intentions before and during the pandemic in Qatar. J Personal Med. (2021) 11:456. 10.3390/jpm11060456 34073655 PMC8225037

[5] Buchan J Catton H. Covid-19 and the International Supply of Nurses: Report for the International Council of Nurses. Geneva: International Council of Nurses (2020).

[6] Kristoffersen M. Does professional identity play a critical role in the choice to remain in the nursing profession? Nurs Open. (2021) 8:1928–36. 10.1002/nop2.862 33715308 PMC8186704

[7] Sabanciogullari S Dogan S. Relationship between job satisfaction, professional identity and intention to leave the profession among nurses in Turkey. J Nurs Manag. (2015) 23:1076–85. 10.1111/jonm.12256 25302666

[8] Tong LK Zhu MX Wang SC Cheong PL Van IK. Nurses who are more willing to participate in the fight against Covid-19: evidence from China. Int J Environ Res Public Health. (2021) 18:7357. 10.3390/ijerph18147357 34299810 PMC8305985

[9] Holland JL Johnston JA Asama NF. The vocational identity scale: a diagnostic and treatment tool. J Career Assess. (1993) 1:1–12. 10.1177/106907279300100102

[10] Slay HS Smith DA. Professional identity construction: using narrative to understand the negotiation of professional and stigmatized cultural identities. Hum Relat. (2010) 64:85–107. 10.1177/0018726710384290

[11] Shun SC. Covid-19 pandemic: the challenges to the professional identity of nurses and nursing education. J Nurs Res. (2021) 29:431. 10.1097/JNR.0000000000000431 33661790

[12] Rasmussen P Henderson A Andrew N Conroy T. Factors influencing registered nurses' perceptions of their professional identity: an integrative literature review. J Cont Educ Nurs. (2018) 49:225–32. 10.3928/00220124-20180417-08 29701865

[13] McNeese-Smith DK Crook M. Nursing values and a changing nurse workforce: values, age, and job stages. J Nurs Admin. (2003) 33:260–70. 10.1097/00005110-200305000-00002 12792281

[14] Zhang Y-D Gao Y-Q Tang Y Li Y-H. The role of workplace social capital on the relationship between perceived stress and professional identity among clinical nurses during the Covid-19 outbreak. Jpn J Nurs Sci. (2021) 18:e12376. 10.1111/jjns.12376 32896954

[15] Zeng Z Wang X Bi H Li Y Yue S Gu S. Factors that influence perceived organizational support for emotional labor of Chinese medical personnel in Hubei. Front Psychol. (2021) 12:2255. 10.3389/fpsyg.2021.684830 34177739 PMC8222904

[16] Correia I Almeida AE. Organizational justice, professional identification, empathy, and meaningful work during Covid-19 pandemic: are they burnout protectors in physicians and nurses? Front Psychol. (2020) 11:566139. 10.3389/fpsyg.2020.566139 33362629 PMC7759469

[17] Sun N Wei L Shi S Jiao D Song R Ma L. A qualitative study on the psychological experience of caregivers of Covid-19 patients. Am J Infect Control. (2020) 48:592–8. 10.1016/j.ajic.2020.03.018 32334904 PMC7141468

[18] Güner Y Turhal E Üçüncüogllu M Tuncel B Akturan S Keleş S. The formation of professional identity in nursing. Türkiye Biyoetik Dergisi. (2021) 8:82–9. 10.5505/tjob.2021.72677

[19] Glerean N Hupli M Talman K Haavisto E. Young peoples' perceptions of the nursing profession: an integrative review. Nurse Educ Today. (2017) 57:95–102. 10.1016/j.nedt.2017.07.008 28755570

[20] Lam SKK Kwong EWY Hung MSY Chien WT. Emergency nurses' perceptions regarding the risks appraisal of the threat of the emerging infectious disease situation in emergency departments. Int J Qualit Stud Health Well-Being. (2020) 15:1718468. 10.1080/17482631.2020.1718468 31975652 PMC7034460

[21] Abad González L Flores-Martos JA Cipriano-Crespo C Pulido-Fuentes M. Strengths and weaknesses of healthcare professionals' identity during the Covid-19 pandemic: a qualitative study within the Spanish context. Soc Sci. (2021) 10:33. 10.3390/socsci10020033

[22] Zhang F Zuo Q Cheng J Li Z Zhu L Li Y. Professional identity during the Covid-19 pandemic: a cross-sectional survey of nurses in China. Am J Crit Care. (2021) 30:203–11. 10.4037/ajcc2021245 33768231

[23] Tong LK Zhu MX Wang SC Cheong PL Van IK. Factors influencing caring behaviour among registered nurses during the Covid-19 pandemic in China: a qualitative study using the Com-B framework. J Nurs Manag. (2022) 2022:1385. 10.1111/jonm.13855 36198011 PMC9874631

[24] World Health Organization. Who Director-General's Opening Remarks at the Media Briefing on Covid-19. Geneva: World Health Organization (2020). Available online at: https://www.who.int/director-general/speeches/detail/who-director-general-s-opening-remarks-at-the-media-briefing-on-covid-19-$-$11-march-2020 (accessed July 11, 2021).

[25] National Bureau of Statistics of China. Preliminary Accounting Results of Gdp for the Fourth Quarter and the Whole Year of 2019. Beijing: National Bureau of Statistics of China (2020). Available online at: http://www.stats.gov.cn/english/PressRelease/202001/t20200120_1724023.html (accessed July 11, 2021).

[26] Ministry of Health. Chinese Health Statistical Yearbook 2019. Beijing: Peking Union Medical College Press (2020).

[27] Matthews J Bialocerkowski A Molineux M. Professional identity measures for student health professionals: a systematic review of psychometric properties. BMC Med Educ. (2019) 19:308. 10.1186/s12909-019-1660-5 31409410 PMC6693256

[28] Takemura Y. The measurements of career identity of nurses and unlicensed assistants at long-term care units. Iryo to Syakai. (2005) 14:83–97.

[29] Zhao H Lu TT Zhang CY. Testing for reliability and validity of chinese version of the nurse's career identity scale. Chin Nurs Manag. (2010) 10:49–51.

[30] Olmos A Govindasamy PJ. Propensity scores: a practical introduction using R. J Multidiscipl Eval. (2015) 11:68–88.

[31] Ren Z Zhang X Sun Y Li X He M Shi H. Relationships of professional identity and psychological reward satisfaction with subjective well-being among Chinese nurses. J Nurs Manag. (2021) 29:1508–16. 10.1111/jonm.13276 33501740

[32] Caricati L D'Agostino G Sollami A Bonetti C A. study on Covid-19-related stigmatization, quality of professional life and professional identity in a sample of Hcws in Italy. Acta Bio-med Atenei Parmensis. (2022) 93:e2022150. 10.23750/abm.v93is2.12613 35545987 PMC9534206

[33] Chemali S Mari-Sáez A El Bcheraoui C Weishaar H. Health care workers' experiences during the Covid-19 pandemic: a scoping review. Hum Resour Health. (2022) 20:27. 10.1186/s12960-022-00724-1 35331261 PMC8943506

[34] Sheng Q Zhang X Wang X Cai C. The influence of experiences of involvement in the Covid-19 rescue task on the professional identity among Chinese nurses: a qualitative study. J Nurs Manag. (2020) 28:1662–9. 10.1111/jonm.13122 32770772 PMC7436396

[35] Ten Hoeve Y Jansen G Roodbol P. The nursing profession: public image, self-concept and professional identity. A discussion paper. J Adv Nurs. (2014) 70:295–309. 10.1111/jan.12177 23711235

[36] Mohammed S Peter E Killackey T Maciver J. The “Nurse as Hero” discourse in the Covid-19 pandemic: a poststructural discourse analysis. Int J Nurs Stud. (2021) 117:103887. 10.1016/j.ijnurstu.2021.103887 33556905 PMC9749900

[37] Li Z Zuo Q Cheng J Zhou Y Li Y Zhu L. Coronavirus disease 2019 pandemic promotes the sense of professional identity among nurses. Nurs Outlook. (2021) 69:389–98. 10.1016/j.outlook.2020.09.006 33077203 PMC7538146

[38] Chen H Li G Li M Lyu L Zhang T A. cross-sectional study on nurse turnover intention and influencing factors in Jiangsu Province, China. Int J Nurs Sci. (2018) 5:396–402. 10.1016/j.ijnss.2018.09.012 31406854 PMC6626264

[39] Lu M Ruan H Xing W Hu Y. The relationship between the participation of nurses in hospital affairs and their level of burnout. J Nurs Sci. (2012) 27:13–5.23107005

[40] Zhao H Zhang CY Lu TT Shi Y Zheng JP Ding Y. Survey on career identity of nurses in 6 class III grade I hospitals. J Nurs. (2011) 18:27-30.

[41] Catania G Zanini M Hayter M Timmins F Dasso N Ottonello G. Lessons from Italian front-line nurses' experiences during the Covid-19 pandemic: a qualitative descriptive study. J Nurs Manag. (2021) 29:404–11. 10.1111/jonm.13194 33107657

[42] Rosser E Westcott L Ali PA Bosanquet J Castro-Sanchez E Dewing J. The need for visible nursing leadership during Covid-19. J Nurs Schol. (2020) 52:459–61. 10.1111/jnu.12587 32779857 PMC7361621

[43] Peng F Tu L Yang Y Hu P Wang R Hu Q. Management and treatment of Covid-19: the Chinese experience. Can J Cardiol. (2020) 36:915–30. 10.1016/j.cjca.2020.04.010 32439306 PMC7162773

[44] Cui S Zhang L Yan H Shi Q Jiang Y Wang Q. Experiences and psychological adjustments of nurses who voluntarily supported Covid-19 patients in Hubei Province, China. Psychol Res Behav Manag. (2020) 13:1135–45. 10.2147/PRBM.S283876 33312005 PMC7727274

[45] Fernández-Castillo RJ González-Caro MD Fernández-García E Porcel-Gálvez AM Garnacho-Montero J. Intensive care nurses' experiences during the Covid-19 pandemic: a qualitative study. Nurs Crit Care. (2021) 26:397–406. 10.1111/nicc.12589 33401340

[46] Crowe S Howard AF Vanderspank-Wright B Gillis P McLeod F Penner C. The effect of Covid-19 pandemic on the mental health of canadian critical care nurses providing patient care during the early phase pandemic: a mixed method study. Intens Crit Care Nurs. (2021) 63:102999. 10.1016/j.iccn.2020.102999 33342649 PMC7832945

[47] Sun M Hennekam S A. Multilevel perspective on the perceived effects of Covid-19 on nurses in China. Empl Relat Int J. (2021) 44:474. 10.1108/ER-10-2020-0474

[48] Deliktas Demirci A Oruc M Kabukcuoglu K. 'It was difficult, but our struggle to touch lives gave us strength': the experience of nurses working on Covid-19 wards. J Clin Nurs. (2021) 30:732–41. 10.1111/jocn.15602 33325080

[49] Moscou-Jackson G Homme M Day J. Non-direct care nurses: professional identity and role in the Covid-19 pandemic. JONA J Nurs Admin. (2022) 52:211–6. 10.1097/NNA.0000000000001133 35348485

[50] Salari N Khazaie H Hosseinian-Far A Khaledi-Paveh B Kazeminia M Mohammadi M. The prevalence of stress, anxiety and depression within front-line healthcare workers caring for Covid-19 patients: a systematic review and meta-regression. Hum Resour Health. (2020) 18:100. 10.1186/s12960-020-00544-1 33334335 PMC7745176

[51] Jun-Hee B Aeyoung S Soo Jung C Sunah P. Influencing the turnover intention of Covid-19 ward and general ward nurses in public hospitals. Korean J Occup Health Nurs. (2021) 30:46–56. 10.5807/kjohn.2021.30.2.46

